# Women’s social care provision in prison has improved but challenges remain: findings from a national survey in England eight years after the 2014 Care Act

**DOI:** 10.1186/s12889-026-27183-w

**Published:** 2026-05-01

**Authors:** Deborah Buck, Adam O’Neill, Nina Marsh, Kate Stalker, Emma Plugge, Claire Hargreaves, Nicola Brimblecombe, Paula Harriott, Catherine Robinson, Jennifer J. Shaw, Katrina Forsyth

**Affiliations:** 1 Social Care and Society, University of Manchester, Manchester, UK; 2 Division of Psychology and Mental Health, University of Manchester, Manchester, UK; 3 Healthwatch England, London, UK; 4 Faculty of Medicine, University of Southampton, Southampton, UK; 5 Social Work, School of Social Sciences, Lancaster University, Manchester, UK; 6 Care Policy and Evaluation Centre, London School of Economics and Political Science, London, UK; 7 Unlock, Maidstone, UK

**Keywords:** Care act, Prison, Social care, Release, Women

## Abstract

**Background:**

At the end of 2024 over 3,500 women were living in prison in England, many of whom have experienced prior trauma and domestic abuse and are more likely than men in prison to self-harm. Compared to women living in the community, they also have higher levels of social care needs, yet little research has been conducted to explore social care provision for this population.

**Methods:**

We conducted surveys of healthcare managers and governors in eleven women’s prisons in England and their corresponding nine local authorities (LAs), to establish how they addressed their responsibilities for women with social care needs eight years on from the 2014 Care Act. Numerical and pre-coded data were analysed in Microsoft Excel using simple descriptive methods (e.g., frequencies, percentages). Descriptive qualitative analysis was used on free-text data.

**Findings:**

The LA survey was completed by 9/9 LA staff; the prison governor survey by 8 staff (representing 10/11 prisons); and the healthcare manager survey by 7/11 staff. Considerable variation was found between establishments in Care Act assessment rates (1% to 36%). Some prisons relied on prison officers or peer supporters who had not received adequate training/supervision to identify social care needs, although all respondents agreed that social care provision had improved since the Care Act. There was less agreement regarding arrangements for transferring assessments between LAs on release. Qualitative analysis provided insight into this and other problems, including identifying women with social care needs; transferring information; gaining access into the prison; and resolving disputes/disagreements between LAs. Several proactive initiatives to improve identification/provision, and promote wellbeing, were described (e.g., regular drop-ins; scoping the use of telecare; linking with external agencies (e.g., neurodiversity and sensory services); an enablement/reablement pathway; and advocacy).

**Conclusion:**

This paper is the first to explore social care provision for women in prison in relation to the 2014 Care Act. Although provision has grown and improved since the implementation of the Act, it is patchy and often suboptimal or “gets forgotten”. Potential ways forward include standardised, flexible screening processes; gender-specific adaptation of screening/assessment tools; and social care training and supervision for officers and peer supporters.

**Supplementary Information:**

The online version contains supplementary material available at 10.1186/s12889-026-27183-w.

## Introduction

As of December 2024, more than 733,000 women and girls were living in prison worldwide, with 3,528 in England [1]. Many women living in prison have experienced trauma, domestic abuse, and/or spent time in care as children [2–4] and are more likely than men living in prison to self-harm [5]. Moreover, women living in prison have higher levels of social care needs than men living in prison or women living in the community [3]. Social care need can include requiring assistance with a variety of activities including activities of daily living; personal care; social interaction and managing relationships; participation in/contribution to society including work and education; and maintaining independence, dignity, and human rights [6–9].

Some individuals who enter prison would have previously been living in the community without their health and social care needs having been identified or addressed [10–14]. In some cases, these unmet needs may be linked to the risk of offending [15–17]. In turn, being released from prison with continuing unmet health and social care needs may perpetuate a cycle of recidivism [18, 19]. Inadequate social care provision is therefore both a public health and a wider societal issue, with implications for the individuals who do not receive the care they need, and for health systems and society as a whole [20, 21]. While prison may offer opportunities for social workers, healthcare staff, prison officers, peer supporters, and/or other staff to identify people within a “captive audience” whose health and social care needs were not adequately addressed while living in the community [17, 22, 23], staff shortages, staff burnout, and inadequate levels of training and awareness among staff, often prevents these opportunities from being harnessed [17, 24].

Whilst there is a growing body of research which shows that social care needs of people living in prison are not adequately met [6, 25–29], this has primarily focused on the male prison estate. There have been fewer studies specifically examining the social care needs of people living in the women’s prison estate [3, 30, 31].

In 2021, the National Women’s Prisons Health and Social Care Review was established by HM Prison and Probation Service (HMPPS) and NHS England [3]. This identified priority areas for women’s social care in prison which included: guidance for, and timely involvement of, social care staff in relation to preparing women for release, including input into resettlement plans and linking with resettlement teams and children’s services where applicable; and the need for consistency in assessments (to include self-referral and referral by family members, as well as health and prison staff). The Review also noted that there are some similarities in the social care needs of people within the men’s and women’s prison estates, but that there are aspects specific to the women’s estate since “women are and have different issues to men in prison” [3] including higher levels of trauma [4]. Moreover, NHS England has posited that the social care needs of women in prison have been “lost to the health agenda” and that this disparity could be addressed by adopting a focused approach to addressing these needs [3].

In England, the Care Act [32], which was implemented in 2015, placed responsibility with local authorities (LAs) (units of local government) for the social care of people living in prison and after their release back into the community. Since then, several surveys have been conducted to explore how LAs have been managing this responsibility [18, 29]. However, the surveys did not include or distinguish the needs of women living in prison.

This work is part of a larger study to explore the nature and extent of women’s social care needs in England’s prison estates. Here, we report findings from three national surveys aimed at (1) prison healthcare managers, (2) prison governors, and (3) their corresponding LAs. The surveys sought to capture the arrangements in place to identify and assess women who may need social care support (as determined by the Care Act) in prison and on release, eight years after the Act’s implementation, as well as how services were being delivered and the challenges faced.

## Methods

We conducted three online surveys of the eleven women’s prisons in England at the time (between January and July 2023), and their corresponding LAs, to establish current practice relating to social care (as determined by the Care Act) in and on release from prison.

### Survey development and content

The bespoke surveys, which were based on a previous national survey of the early arrangements that LAs had put in place to meet the social care needs of adults living in the wider prison estate in England [29], were developed for completion by prison healthcare managers, prison governors, and their corresponding LAs. The original survey tool was developed by the previous study’s authors [29], piloted with a small number of LAs in the north-west of England, and revised accordingly [29]. The survey contains a combination of numerical questions, pre-coded scales, and free-text questions (see Supplementary Files S1 to S3), and sought details of how authorities/establishments were meeting their responsibilities regarding social care for women living in prison (including identification, assessment, care planning, and provision); social care on release from prison; and the challenges that authorities/establishments may have encountered in meeting their obligations under the Care Act. Most questions were the same across the three surveys, but others were specific to respondent type (see Supplementary Files S1 to S3). In particular, a question asking whether and how screening tools used for women differed from those used for men living in prison was added to the survey for healthcare managers.

### Survey distribution

LAs with a women’s prison in their area were identified and the Director of Adult Social Services was contacted by the research team via email. The research team obtained up-to-date names and contact details for all LA managers, details for prison governors from HMPPS, and details for healthcare managers from NHS England. Invitations and cover letters detailing the purpose of the survey were distributed via email through a local gatekeeper in January 2023. Subsequently, the survey was distributed electronically and included a direct email address for the research team. The surveys were sent to the prison governors and prison healthcare managers of the eleven women’s prisons in England and to senior managers/social care leads for prisons at the nine corresponding LAs in which these prisons were situated. Surveys were hosted on Qualtrics (University of Manchester approved), though respondents were also given the option to return a Word document version via email. Data collection closed in July 2023.

### Data analysis

Data from the LA, healthcare, and governor surveys were stored in three Microsoft Excel (version 2307) spreadsheets. Numerical and pre-coded data were analysed in Excel using descriptive methods (i.e., frequency counts and percentages where applicable). We used descriptive qualitative analysis on the free-text data [33]. Three researchers first familiarised themselves with the data for each free-text question, in the Excel spreadsheets. Two researchers then independently coded the data in relation to the individual question (e.g., ‘reception screen’, ‘self-referral’, or ‘healthcare staff’ within the ‘identifying needs’ question). A third researcher checked and consolidated the coding for each question, to ensure agreement and reliability, before drafting the findings. A fourth researcher then reviewed the findings against the raw data, to ensure they presented a fair reflection of participants’ responses. Comparisons between groups (i.e., LAs, healthcare, and governors) were not undertaken on either the quantitative or free text data, given the small sample sizes and inconsistent level of detail provided respectively.

## Findings

**Fig. 1:**
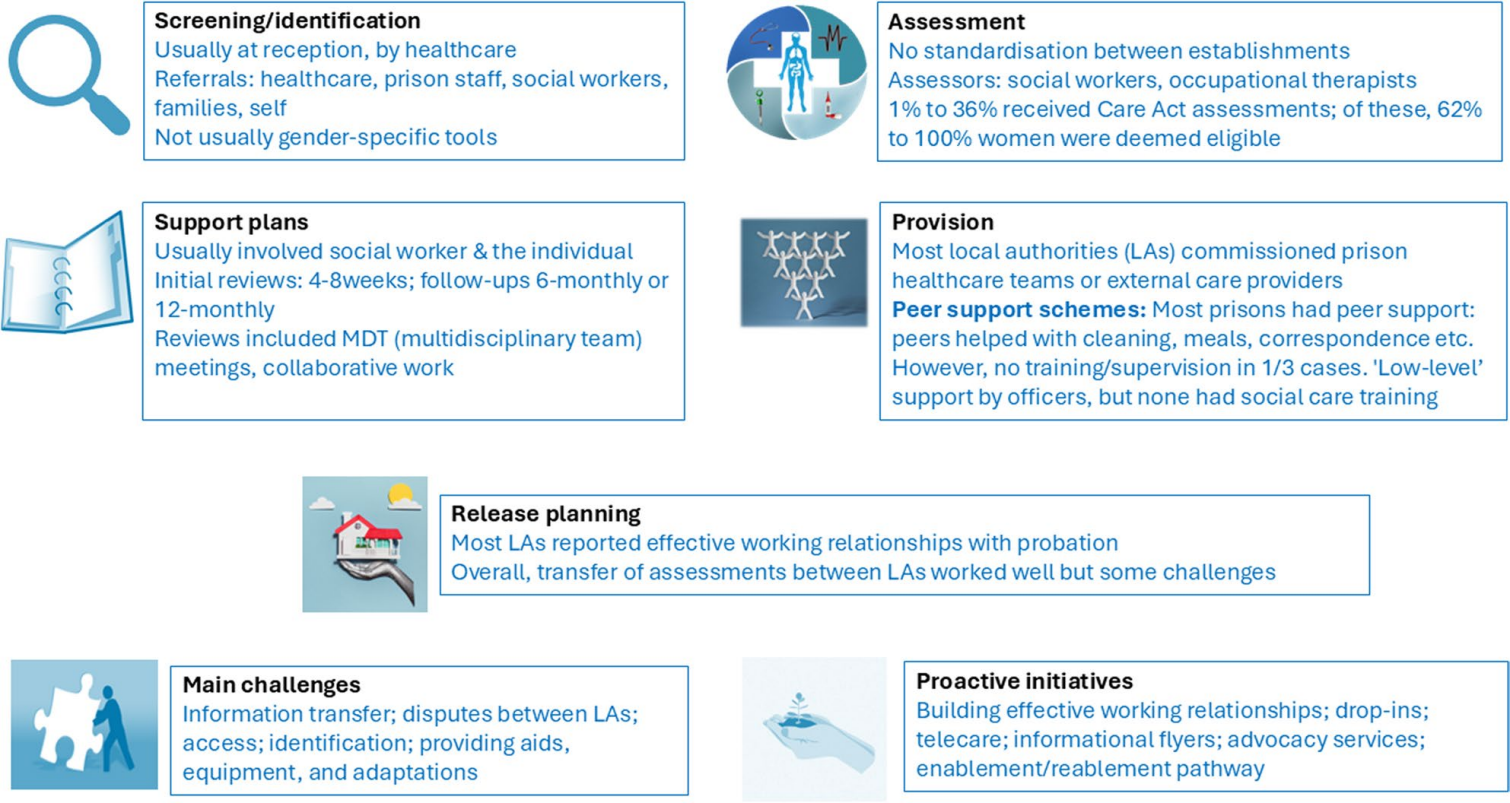
Summary of key findings

The LA survey was completed for all nine of the LAs containing women’s prisons in England. The prison governor survey was completed for 10 of the eleven women’s prisons. In two cases, the governors were responsible for two prisons; therefore, there are eight prison governor respondents for this survey. The healthcare survey was completed for seven of the eleven women’s prisons. The key findings from all 3 respondent groups are described in two main sections: (a) social care in prison, and (b) social care on release from prison. We provide anonymised illustrative quotes where appropriate. A summary of the key findings is provided in Fig. 1.

### Social care in prison

#### Identifying social care needs in prison

All three surveys included a free-text question asking how women with social care needs in prison were identified. The most common mechanism for identification was at reception screening on entry to custody, usually undertaken by the healthcare team, although identification by prison officers, safer custody teams, and/or during prison induction was also mentioned.

Aside from reception screening, some respondents added that any member of staff could make a social care referral should a need arise at any time during a resident’s stay, although referral routes nevertheless varied between establishments. Some respondents listed one or two, while others referred to a wider net from which referrals were received. Referral routes included physical and mental healthcare teams, prison staff (e.g., prison officers, Safer Custody teams, offender managers, Liaison and Diversion teams), social workers, external partner agencies including third sector organisations and other LAs, families, and self-referrals. Self-referral tended to be through completion of an ‘app’ (a written application form) that was sent to the LA via the prison healthcare team. One LA described their system as “not great” (LA 7), noting a heavy reliance on prison staff to identify women living in prison with social care needs.

The healthcare survey asked respondents to describe whether and how the tools they used for women differed from those used for men living in prison. Most healthcare managers stated that their establishment used generic tools rather than tools specifically designed for use with women living in prison. However, one respondent reported that the Offender Management Unit’s Basic Screening Tool 1 had been “tailored to women’s services” (HC 1), although it was not clear how.

There were examples of proactive initiatives aimed at improving the identification of women with social care needs. This included the building of good working relationships between the LA and the prison via link workers (forming links with offender management units, inclusion teams, and healthcare teams); social care ‘drop-ins’ with Prison Social Care Practitioners from the LA; and the distribution of flyers about social care to improve general awareness.

#### Assessing social care needs in prison

The LA survey included a free-text question about how the social care needs of women living in prison were assessed and, where specified, by whom.

Where details of the assessment tools used were provided by respondents, this was often described in broad terms as being based on the Care Act’s eligibility criteria. One LA referred to the ‘Trusted Assessor’ document that was completed by healthcare staff to identify individuals’ social care needs and the level of required support. The respondent explained that the Trusted Assessor document was to be used under specific circumstances, for example, in the event of a health crisis to enable prompt assessment before a full assessment could be arranged. In another example, a strengths-based approach was reported as being adopted.

In terms of who carried out assessments, social workers and occupational therapists were usually specified, mostly employed by the LA rather than other agencies. Again, there were indications of good social care practice where several LAs reported having dedicated prison practitioners or specialist teams. One LA reported a wider pool of specialised staff who conducted assessments, allowing them to respond more sensitively to a variety of needs. This LA explained the role of each specialised staff member, with specialist sensory workers assessing visual and hearing-impaired individuals; an OT care manager assessing for equipment to aid social care needs; and a forensic social worker assessing women who had been in contact with mental health services. Another LA emphasised the value of free access to and movement around the prisons for staff carrying out assessments, enabling them to do so independently without a chaperone, once key training and vetting had taken place.

#### Number of social care assessments undertaken

Each of the three surveys included questions about the numbers of women assessed and the numbers deemed eligible for LA social care support under the Care Act over a given three-month period (July to September 2022). Data for these questions was missing or recorded as ‘not known’ for at least 50% of respondents to the healthcare and governor surveys. This section therefore reports LAs’ responses only, for which there was a 100% response rate to these questions. The estimated number of assessments conducted ranged from 4 to 260 (see Table 1). Proportionate to the number of women living in prison within the LAs’ boundaries, this suggests that between 1% and 36% of women had received a Care Act assessment, with the overall average being 13%. Between 60% and 100% of those assessed were subsequently deemed eligible for social care support.

**Table 1:** Care Act assessments by LAs over a three-month period

Authority	Women assessed *n*	Women deemed eligible *n* (%)	Women living in prison within LAs’ boundaries (Sept 2022) *n*	Proportion (%) assessed based on no. of women living in prisons within LAs’ boundaries***
1	56	45 (80%)	350	16%
2	4	4 (100%)	230	2%
3	4	4 (100%)	79	5%
4	260*	195 (75%)*	724	36%
5	5*	5 (100%)*	433	1%
6	9*	9 (100%)*	356	3%
7	13	8 (62%)	291	4%
8	28**	Not reported	245	11%
9	30*	30 (100%)*	293	10%

^*^As estimated by the responding LA, ^**^Response was 55 in last 6 months, ^***^Calculated by the authors

#### Development and review of social care and support plans

This section summarises responses to free-text questions, which were included in the LA and healthcare surveys, about who was involved in developing social care and support plans, and what arrangements were in place to review them.

In some cases, respondents did not specify the professional involved in developing social care plans. Where specific staff were mentioned, this was most likely to be a social worker. Collaborative work with the LA was emphasised in many of the responses from healthcare teams. For example, regular multidisciplinary meetings and reviews of patients to facilitate co-working, including the reviewing and updating of care plans as required, were highlighted. Responses also indicated that the women themselves were usually involved in their care planning. The LA respondent who referred to the ‘Trusted Assessor’ document for interim assessment noted that this included a brief support plan, which was ratified by the LA, and that this could lead to a more in-depth Care Act assessment and support plan.

Reviews of initial care plans were reported to occur between four and eight weeks after development, with subsequent follow-ups being either six-monthly or annually. In some cases, reviews took place face-to-face, whilst others were completed via telephone or virtual meeting. The involvement of multidisciplinary teams was mentioned by two LA respondents. Overall, a flexible picture emerged regarding follow-up reviews in response to changes in need or direct requests for the care plan to be reviewed. Some LAs highlighted the importance of maintaining an ongoing relationship with the resident once care needs were being met, to ensure that if their needs changed, the service would be able to respond accordingly.

#### Provision of social care in prison

This section focuses on how LAs were providing social care to women living in prison. Most LAs either commissioned prison healthcare teams or external care providers. One LA provided the service themselves, and another reported having no prearranged commissioning, clarifying that all requests so far had been for equipment and advice, but that they had the budget to provide social care in prison if required.

Some respondents added that prison officers or peer supporters would provide ‘low-level’ support (e.g., assisting with daily living activities or other non-intimate needs that did not meet the Care Act’s threshold for personal care). However, responses to the prison governor survey indicated that there had been no social care training for prison officers in any of the establishments. Moreover, provision of training and supervision for peer supporters was variable, as described in the next section.

#### Peer support schemes in prison

Each of the three surveys included a section about peer support schemes. Almost all respondents stated that peer supporter/buddy systems were operational in their establishment. However, in some cases, no formal training was available for peer supporters, or there was uncertainty regarding the type and level of training provided (see Table 2).

**Table 2:** Peer support systems in women’s prisons

Survey	Peer support systems implemented *n*	Training for peer supporters *n*	Supervision of peer supporters *n*
LA (9 respondents)	7	4	3
Governors (8 respondents)	7	5	2
Healthcare (7 respondents)	6	4*	4**

^*^Plus, one unsure whether formal or not, ^**^Plus, one unsure

When known, training was reported to be carried out by a variety of staff, including healthcare, social work teams, a third-sector organisation, or induction orderlies. One LA respondent explained that a Disability Liaison Officer coordinated internal peer supporter training and aimed to formalise the training package into different workbooks to ensure comprehensiveness. A list of topics that may be covered in peer supporter training, based on additional information provided by LA respondents and prison governors, is available in Supplementary Table S1.

Some respondents described the forms of social care support that peers provided. This included collecting meals, laundry, cleaning, and support with correspondence. One respondent reported that staff completed a one-page profile/contract with peer supporters outlining the tasks they were expected to complete and clarifying that they should not do anything that was not listed. These contracts would be signed and reviewed on an ongoing basis.

As shown in Table 2, even where training was provided for peer supporters, not all received supervision. In cases where supervision was provided, this was from a range of staff, including the Disability Liaison officer, social care, prison officers, the Safer Custody Team, or the Custodial Manager of Equality and Diversity.

#### Links with children’s social services

This section reports findings from the LA survey in which respondents were asked to describe their links with children’s social services. One LA reported having a good, direct relationship with children’s social services, which had been developed through their memorandum of understanding (MoU). The MoU included details on being aware of the needs of mothers and pregnant women living in prison. Other LAs reported that links with children’s social services were used as and when necessary, or that they had not yet needed to link with them. However, these respondents often clarified that the needs of mothers in prison with social care needs were nevertheless considered. For example, LAs would ensure that they arranged and maintained contact between mother and child, or link in with Leaving Care Workers for any young residents who were care leavers.

#### Improving wellbeing

LAs and prison governors were asked to detail how they worked to improve the general well-being of women living in prison (to prevent, delay, or reduce deterioration in social care needs), as specified under the Care Act. Responses from LAs revealed a range of diverse and innovative avenues to promote women’s wellbeing in prison. Examples included holding regular drop-ins; working with a university to promote physical health and movement; scoping the use of telecare; visiting women regardless of whether they had current care and support needs or not; adoption of an enablement/reablement pathway for those needing strengths-based, goal-focused, short-term intervention; working alongside education to get people “linked in”; working with the prison to make reasonable adjustments; working with the safeguarding adults board to better understand safeguarding in prison; prescribing low-level equipment/aids; linking in with external agencies (e.g., neurodiversity and sensory services) who provided support in the prison setting; and piloting of advocacy services.

Similarly, responses from prison governors indicated many positive initiatives including the provision of a sensory room providing a “safe soft environment” for promoting mental health wellbeing (PG 1); the Diversity and Inclusion team “working closely with a proportion of the population to promote general wellbeing” (PG 1); healthcare health promotion; a Wellbeing Team focussing on purposeful activities on the wing and promoting social integration; “a schedule of on-unit activities supported by an occupational therapist to promote integration and wellbeing or prisoners” (PG 1); purposely refurbished “Social Care cells” (PG 4); and in-cell activities and support from education providers. Several respondents referred to the role that regular safety meetings and multidisciplinary team meetings played in promoting overall wellbeing, while two also referred to the contribution of peer support.

### Social care on release from prison

#### Identifying social care needs pre-release

Each of the three surveys included questions about identifying individuals with social care needs on or following release from prison. Most governor survey respondents stated that this was handled by the LA and provided no further details. Therefore, we report the LA and healthcare survey responses only. Respondents to the LA survey reported that notifications and referrals for release planning assessments came from various sources, including healthcare, offender managers, inclusion, care coordinators, prison resettlement teams, parole boards, third-sector community organisations, or self-referral. Some respondents clarified that referrals could be for individuals not currently under their care where there was any concern about how they might manage once living back in the community, and one emphasised the importance of having “good links” (LA 9) with the prison, healthcare, and inclusion, whereby they worked together to identify people who may need support on release. Attendance at weekly pre-release meetings was also mentioned as a way of identifying who may require such support.

Like LA survey respondents, healthcare staff referred to working with external agencies including third sector organisations. In addition, reference was made to release clinics and liaison with OMU regarding pre-release planning. One healthcare respondent noted that individuals with “complex needs, concerns or difficulties” were discussed at weekly multidisciplinary Safety Intervention meetings (HC 7), while another pointed out that “planning for release will begin at the earliest opportunity” (HC 3) and that patients are reassessed if there are any changes in health status.

#### Transferring assessment between authorities

All respondents were asked to rate how well the process for transferring prisoner assessments to the receiving authorities, when individuals were being released from prison, were working. As can be gleaned from Table 3, the process was most often described as working ‘fairly well’. There were, however, some challenges (outlined below).

**Table 3:** Transferring assessments between LAs on release from prison

	LA survey (***n*** = 9)	Healthcare survey (***n*** = 7)	Governor survey (***n*** = 8)
Very well	0	1	2
Fairly well	7	4	3
Not very well	0	1	2
Not at all well	2	0	0
Unsure/missing	0	1	1

#### LAs’ partnerships with probation services

Most LAs reported a positive, effective working relationship with probation, although one respondent highlighted a lack of engagement and information-sharing from probation services. However, they added that their authority was currently working with probation to improve knowledge with providers in the community about the roles and responsibilities of managing individuals with offending histories. Further challenges relating to release are included below.

### Perceived challenges to delivering Care Act reforms

All respondents were asked to select three out of sixteen responses regarding the main challenges that their authority or establishment was encountering in delivering the Care Act reforms for women living in prison. This included an option for respondents to describe any challenges not listed in the multiple-choice list. Full survey questions are provided in Supplementary Files S1 to S3.

The main challenges reported by respondents to the LA survey were ‘transferring information between LAs’; ‘identifying eligible prisoners’; and ‘providing aids, equipment, or adaptations’. In addition, one respondent referred to challenges regarding the lack of staff available to go into the prison, noting that the “prison side often gets forgotten” (LA 7), while another noted poor understanding within the prison of what the Care Act can provide, and poor communication from the prison (LA 1). From the healthcare team perspective, ‘Providing care services in prisons’ emerged as the key challenge, although in three cases this question was skipped. In addition, one respondent to the healthcare survey mentioned the impact on Primary Care Services’ resources when overnight personal care was required, though they did not provide further detail (HC 1). The two most common challenges chosen in the governor survey were ‘identifying eligible prisoners’ and ‘providing aids, equipment, or adaptations in prison’. However, one respondent (PG 7) wrote that, despite having a Memorandum of Understanding in place with the LA, “social care does not exist in the prison due to lack of engagement by [LA name]”, explaining that social care issues were being managed by the prison and that payment for equipment was inappropriately taken from the prison budget.

#### Specific challenges for release-planning

This section focuses on insights from free text responses regarding problems with the transfer of assessments between LAs, with particular emphasis on the consequence for release-planning.

LA survey respondents noted that the receiving authority sometimes disagreed with the assessments made by the prison, declined referrals for individuals with social care needs moving into their area, or disputed claims around ordinary residence.

“Often local authorities do not understand that they have a duty to assess the person moving into their area and will decline to take the referrals” (LA 9).

“Local areas refusing to accept persons even when they have been previously known and expect the prison area to pick everything up though it is not their home area or Ordinary Residence …. many LA are difficult.” (LA 4).

Ordinary residence for an individual leaving prison is presumed to be the area in which they were ordinarily resident before the start of their sentence, although this is subject to the wishes of the individual, who may have restrictions imposed on where they may live or may wish to move elsewhere [34]. However, it was noted that when the receiving authority contained a prison, then the transfer of assessments went “much smoother” (LA 3).

One LA respondent stated that many women with social care needs were not referred for assessment prior to release because their needs were being met by the regime while living in the prison. This authority was actively working to ensure they received “the right referrals at the right time” (LA 5) and was “keen to try and break some of the cycles that occur for some of the women by assessing and sharing information when release occurs” (LA 5). Similarly, another LA respondent stated that they recognised that it is often the case that the prison regime meets some social care needs, but this is taken into consideration when carrying out pre-release assessments. A further problem highlighted by LA respondents was a lack of feedback, for example, not knowing whether an assessment made by the transferring authority had been received and/or acted upon by the receiving authority.

Housing allocation upon release was raised as another problematic area, raised by respondents to the LA and healthcare manager surveys. This was said to be due to, for example, expectations that the prison would independently ensure that appropriate residence allocation was arranged; or assessments for individuals with complex needs not being accepted within an appropriate timeframe.

“Assessments for people with more complex needs are not accepted within a timely manner to enable support to be put in place for release.” (LA 1).

“Often if patients require housing …. there are delays in local authorities accepting responsibilities.” (HC 1).

Similar issues of timing were echoed in the healthcare and governor surveys. For example, one healthcare respondent referred to delays in LAs accepting responsibility to arrange housing, while a respondent to the governor survey expressed concern that delays led to increased social care needs and noted a lack of support from their LA.

“The referral process is in place however the community engagement /acceptance of assessments takes too much time which can cause deterioration. Main issues: Timescales, community social workers being allocated and supporting with release planning in the prison.” (PG 6).

Finally, another prison governor survey respondent noted the challenge of “Local Authorities taking responsibilities for prisoners with care needs if they are moving to a new area on release.” (PG 1).

### Perceived impact of the Care Act

All respondents were asked to what extent they agreed with statements regarding improvement in social care in their authority/establishment since the implementation of the Care Act. As shown in Table 4, each respondent who answered the question either completely or somewhat agreed that social care had improved in their authority/establishment both in custody and on release.

**Table 4:** Perceived impact of the Care Act

**Statement:**	**Survey:**	**Completely agree**(***n***)	**Somewhat agree**(***n***)	**Somewhat disagree**(***n***)	**Completely disagree**(***n***)
The implementation of the Care Act has improved the social care of prisoners in custody in this authority/establishment	LA	7	2	0	0
	Healthcare*	1	5	0	0
	Governor*	5	2	0	0
The implementation of the Care Act has improved the social care of prisoners on release in this authority / establishment	LA	5	4	0	0
	Healthcare*	1	5	0	0
	Governor*	3	4	0	0

^*^Data missing n=1

### Perceived quality of social care provision

Respondents to each survey were asked to what extent they agreed with statements regarding how good social care was in their authority/establishment. As shown in Table 5, most respondents felt that the social care of people living in or released from prison was good at least to some degree, although there was some disagreement around this issue.

**Table 5:** Perceived quality of social care provision

**Statement:**	**Survey:**	**Completely agree**(*n*)	**Somewhat agree**(*n*)	**Somewhat disagree**(*n*)	**Completely disagree**(*n*)
The social care of prisoners in custody is good in this authority/ establishment	LA	5	4	0	0
	Healthcare	4	3	0	0
	Governor*	3	3	1	0
The social care of prisoners on release is good in this authority/ establishment	LA	4	5	0	0
	Healthcare	2	4	1	0
	Governor*	2	4	1	0

^*^Data missing n=1

## Discussion

### Summary of key findings and comparison with the literature

This is the first national survey focusing on the identification and provision of social care needs specifically within the women’s prison estate in England. All nine LAs with women’s prisons in their area responded to the survey which showed that the estimated proportion of women living in prison receiving a Care Act assessment varied across LAs, from 1% to 36%. Whilst the current study cannot explain these variations or determine whether all those with social care needs were indeed assessed, there are several possible explanations including varying levels of experience, pro-activeness and/or differences between staff and prison populations across authorities. For example, the site with the highest proportion of Care Act assessments (Site 4) contains two women’s prisons as well as a particularly high number of establishments in the male prison estate. This site is therefore perhaps more familiar with the Care Act as it applies to the prison setting, which may partially explain why it had the highest assessment rate. This site also has the prison with the highest proportion of ‘older women’ with 26% of women aged 50 or over (as of March 2025) which may also be a contributing factor. Nevertheless, the contrast across the women’s estate in the proportion of assessments may be a cause for concern, and suggests that in some prisons, women’s needs may not be being identified. A survey conducted in 2016 [29], which included all LAs containing prisons in England, found that almost 1,800 people living in prison were identified as having social care needs in the first year since the implementation of the Care Act. Of these, 87% received an assessment and 50% of those assessed were deemed to have met the eligibility criteria for social care. However, it also revealed marked differences in referral rates between similar types of prisons (albeit the survey captured data soon after the Care Act came into force and would have primarily reflected the male prison estate).

Many women entering or living in prison have or are experiencing trauma [4] and may not be aware of their rights nor be in a place emotionally to voluntarily disclose any needs for social care. Moreover, almost 60% of custodial sentences given to women are short sentences (i.e., for less than six months) [35]. This means that their needs may not be identified and/or they have less opportunity to access services than those on longer sentences; this is also the case for women on remand [3]. Missed opportunities to address unmet needs among women prior to their release from prison may prove disastrous when services fail to engage, particularly for people who have not been equipped with the life skills to cope once living back in the community or effectively advocate for themselves. This in turn reduces the likelihood that the ‘revolving door’ issue can be broken, i.e., women will remain trapped in a cycle of offending, trauma, poverty and discrimination [36].

Despite these concerns, respondents felt that social care had improved in their authority/establishment both in custody and on release, since the Care Act 2014. There were several examples of proactive and good practice, such as instances of multidisciplinary working (e.g., LAs building good relationships with probation and children’s services, and/or with the prison via link workers). However, although the surveys did not ask about different approaches to care, no mention was made of trauma-informed practice despite the introduction in 2015 of the ‘Becoming Trauma-Informed’ initiative for women’s prisons in England and Wales [37]. This is particularly crucial in this context, given that a high proportion of women have experienced serious traumatic events prior to entering prison, such as childhood trauma, domestic abuse, homelessness, and/or mental health problems [2]. Moreover, custodial sentences can themselves be traumatising and/or lead to re-traumatisation [38] and can place immense strain on women’s ability to care for their children, grandchildren, and/or wider family [2].

There appeared to be little gender-specific screening for social care needs. This is cause for concern as it suggests that issues which are more prominent among women, such as childcare responsibilities, domestic abuse, and fear of social services [3], are not being considered when women undergo screening for social care needs.

Another important aspect of social care in prison is the provision delivered through peer support schemes, known to fill an important gap in meeting social care needs [22]. Such schemes were present in most women’s establishments, but a third of prisons in our study offering peer support schemes did not provide training or supervision. The lack of formal training and supervision is in accord with recent literature [22, 25, 26, 39–41] and is concerning on several levels. Inadequate training and supervision place peer supporters and/or those receiving support at risk of exploitation or inadequate support [42–44], and burnout [45, 46], and lack of supervision can leave peer supporters feeling unsupported [22]. Previous studies have highlighted the benefits of adequate training for peer supporters including development of empathy for the individual while understanding limits of the role in “ambiguous interpersonal situations”, thereby knowing when to ask for help [47]; peers becoming better able to identify people in need of social care and support on release including a greater understanding of the referral process [18]; improved relationships with staff which can result in officers becoming more willing to work collaboratively with peer supporters [42, 47]; and promotion of dignity and respect within the prison population [44]. These findings highlight the need for formal training and supervision of peer supporters in prison. This should include trauma-informed care/support, which survey responses suggest is not currently covered even in prisons where training is provided.

Although the transfer of social care assessments between LAs was reported as working ‘fairly well’ in most cases, there were several potentially surmountable barriers to effective release-planning. For example, some receiving LAs challenged or refused referrals, and/or there were delays in information transfer. Similarly, a survey of 86 LAs conducted in 2020 [18], which covered both men’s and women’s establishments, noted disputes over the respective responsibilities of sending and receiving authorities and problems with the timely sharing of information, while an international scoping review of social care on release from prison [48] reported that strong inter-agency relationships and shared understanding and ownership were key to effective community support upon release. The review also identified examples of good practice, including the development of specialist prison social worker roles and personalised pathway documents.

Our survey findings align with the priorities identified in NHS England’s review of health and social care in women’s prisons [3] (as described in the Introduction). For example, the variety of referral routes described by different LA and healthcare respondents, and the challenges described regarding release-planning. We found that a further challenge to release planning was that, since many women were reportedly having their social care needs met by the prison regime, they were not referred for social care assessment prior to release. Likewise, Hargreaves et al. [18] and Tucker et al. [48] reported a lack of systematic processes to identify those needing social care on release from prison, particularly those whose needs had previously been met by the regime or may only emerge once living in the community. While these problems around social care on release are not specific to women’s prisons, there are far fewer women’s prisons in England, meaning that most women are situated a long way from home and, usually, from the place they wish to return, adding further to the difficulties and anxieties around release planning and ensuring their needs are met.

### Strengths and limitations

This is the first study to systematically examine the provision of social care for women living in prison in England. Survey response rates were high, with all relevant LAs and all but one governor responding to the survey, although response to the healthcare survey was less favourable, representing seven of the eleven women’s prisons. This may have introduced some bias into the findings. In particular, healthcare managers were the only participants to be asked about gender-specific tools (given the nature of their role), and we cannot determine whether the four of the eleven who did not respond had adapted their screening tools accordingly.

While the overall response rate to the survey was very good, with rates of 80% or higher being considered excellent [49], some individual questions were left unanswered in the healthcare and governor surveys, particularly items relating to the number of Care Act assessments completed and the number of women deemed to meet the eligibility criteria. For this reason, we reported assessment rates based on LA survey data only. However, this is likely to be the most accurate given that Care Act assessments are the responsibility of the LAs.

Some respondents estimated rather than sought exact figures for key data such as the proportion of women living in prison identified as having social care needs. The surveys did not ask respondents to identify any specific social care needs that women might have in comparison to men living in prison and did not enquire about needs around any caring responsibilities for children. These issues were, however, explored as part of one-to-one interviews with women within another part of this study. While trauma-informed practice is not explicitly covered as part of the Care Act, the safeguarding of adults is, and it may have been valuable to have explored this within our survey.

The small sample size and the inconsistent level of detail provided in the surveys’ free-text questions meant it was not meaningful to make between-group comparisons. Finally, our survey did not include questions explicitly asking about perceived strengths of the arrangements that participants had in place.

### Implications for policy and research

It is important to ensure that women are aware of their social care rights and the ability to self-refer for assessment. However, some LAs expressed concern that prison officers were sometimes relied upon to identify/refer women who may require social care support. Given that all eight respondents to our prison governor survey reported that the prison officers in their establishments had not received training in social care, this should be a priority. This could begin with simple awareness-raising strategies such as posters/flyers and embedding social workers in multidisciplinary team meetings. However, it should also be acknowledged that the high turnover of staff in the prison system presents its own challenges, with recent official statistics reporting a leaving rate of 12.5% amongst band 3–5 prison officers for the 12 months ending 31 December 2024 [50]. High turnover also means that staff are less likely to be knowledgeable about and have a rapport with individual residents, and therefore not as sensitive to changes in their behaviour and needs nor to have built the trusting relationships that are seen as important to assessing needs.

While there appeared to be a strong perception among respondents that social care had improved since the implementation of the Care Act, challenges were highlighted that should be addressed in future policy initiatives. Improvements need to be made to ensure easier access to the prison for social care providers; to the timeliness of information transfer between LAs; to relationship building with external agencies including other LAs; and to identifying people with social care needs particularly pre-release when needs may have been met by the prison regime and/or peer support systems, rather than through a formal support package.

Some degree of variation in the proportion of women receiving assessments under the Care Act might be expected, given that women’s prison populations are not necessarily equivalent across authorities, and we know some establishments house a higher proportion of older women than others, but it is also important to note that social care needs are not limited to the older prison population: younger residents with physical or mental health issues, substance use, or learning disabilities may also require social care [8]. However, excluding the site containing the prison with the highest proportion of women aged 50 or over, there remained a sizeable variation (from 1% to 16%) in assessment rates, and further research is warranted to explore these differences, and to determine the extent of unmet social care needs among women both while living in prison and upon release to the community. It is of note that official data on social care needs of people living in prison is lacking, particularly post-release [48]. While acknowledging that women’s prisons in England make up a small proportion of the entire estate, more research is needed generally to understand this specific population. Indeed, as part of our wider study, we are analysing data from qualitative interviews with staff and women living in the women’s prison estate, which we envisage will provide much needed, valuable insights into the social care needs of this vulnerable group.

Over half of women in prison are sentenced to immediate custody for less than six months and for minor crimes [51, 52]. Our ongoing work suggests that social care needs are not very well met in the prison setting, and short sentences may further impede the provision of adequate assessment and support. Further research around this issue would therefore be valuable. However, the 2025 Independent Sentencing Review has recommended that the Government legislate to ensure short custodial sentences are used only in ‘exceptional circumstances [53].

## Conclusion

The findings from this study help to bridge the knowledge gap regarding how women’s social care needs are being addressed in prisons across England and offer unique empirical data representing this population. The survey confirmed a lack of standardisation in screening and assessment processes despite the small number of women’s establishments and highlighted a lack of gender-specific screening and assessment tools. Imprisoned women are a vulnerable population, with many likely to have significant social care needs. It is therefore crucial to ensure that these needs are addressed effectively when they are in prison. The reliance on prison officers and peer supporters to identify these needs in an informal manner points to the need for greater investment in training to raise awareness about issues around social care among staff and peer supporters, which in turn will help to improve women’s outcomes including their physical and mental wellbeing. Other potential ways forward include a more standardised but flexible screening process, which includes active case finding throughout; gender-specific adaptation of identification and assessment tools; and collection of data to monitor the number of women with social care needs in and on release from prison.

## Supplementary Information

Supplementary Material 1. Local authority survey questions.

Supplementary Material 2. Healthcare manager survey questions.

Supplementary Material 3. Prison governor survey questions.

Supplementary Material 4. Topics covered in peer supporter training.

## Data Availability

The datasets used and/or analysed during the current study are available from the corresponding author on reasonable request.
