# Supplementary material for: Women’s social care provision in prison has improved but challenges remain: findings from a national survey in England eight years after the 2014 Care Act
Source: BMC Public Health. 2026 May 1;26:1930. doi: 10.1186/s12889-026-27183-w (PMC13285503; doi:10.1186/s12889-026-27183-w)
Supplement: Supplementary file 2 — Supplementary Material 2. Healthcare manager survey questions. [file 12889_2026_27183_MOESM2_ESM.docx]

**Supplementary File S2. Healthcare manager/provider survey**

**Women’s Social Care in Prison:**

**Identifying needs and an appropriate response**

**Healthcare Provider Survey**

**(Confidential Information)**

**Please tick this box**  ☐  **to confirm that you have read the Participant Infromation Sheet; and that by completing this questionnaire you consent to your data being used for the purposes of the study.**

**The personal information we collect and use to conduct this research will be processed in accordance with UK data protection law as explained in the Participant Information Sheet and the Privacy Notice for Research Participants.**

**YOUR DETAILS**

| Name of person completing the survey: | Click here to enter text. |
| --- | --- |
| Role of person completing the survey: | Click here to enter text. |
| Name of authority: | Click here to enter text. |
| Contact email in case of queries: | Click here to enter text. |
| Signature of person completing the survey: | Click here to enter text. |

By signing this consent form, I declare that I have provided accurate identity details (full name and electronic signature).

**Please try to avoid using information which may identify other individuals in your answers.**

**SECTION 1: SOCIAL CARE IN WOMEN’S PRISONS**

| **Please outline how your establishment is identifying prisoners with social care needs,** including (i) who undertakes this activity (e.g. social work staff; prison healthcare staff; prisoner orderlies) and (ii) how such prisoners are identified (e.g. via a formal screening tool on prison entry; through annual reviews). |
| --- |
| Comment |

| **Please describe how your establishment is assessing prisoners with social care needs,** including (i) who undertakes these assessments (e.g. prison social workers, locality team social workers, other prison staff, third sector organisation staff) and (ii) any standardised assessment tools used (e.g. FACE Overview Assessment). |
| --- |
| Comment |

| **Please describe how your assessment tools differ for men and women** (if applicable) e.g. pregnancy, childcare |
| --- |
| Comment |

| **Please detail the arrangements your establishment has put in place to develop care and support plans for prisoners with eligible social care needs,** including (i) who undertakes this activity (e.g. prison social workers; locality team social workers; other prison staff; third sector organisation staff) and (ii) how prisoners themselves are involved. |
| --- |
| Comment |

| **Please describe the arrangements your establishment has put in place to review prisoner’s social care needs,** including (i) follow-up assessments (ii) reviewing care plans |
| --- |
| Comment |

| **Please describe the arrangements your establishment has made to provide/ procure services for prisoners with eligible social care needs,** including (i) who delivers this support (e.g. prison officers; prison healthcare assistants; local authority-employed social care workers; third sector staff; prisoner helpers) and (ii) their base (e.g. prison or the community). |
| --- |
| Comment |

| **Please describe any processes your authority has in place for prisoners who act as a carer,** including (i) if carer's assessments are undertaken, (ii) if so, who undertakes these assessments (e.g. prison social workers, locality team social workers, other prison staff, third sector organisation staff) and (iii) any standardised assessment tools used (e.g. Full assessment, 'light touch' assessment) |
| --- |
| Comment |

| **Does your establishment have a peer carer/ buddy system?** *If no, proceed to next question* |
| --- |
| Yes ☐ No ☐ |
| **Do the peer carers/ buddies receive formal training?** |
| Yes ☐ No ☐ |
| **If yes, please describe (e.g. Who provides the training? How long does it last? What is included?)** |
| Comment |
| **Do the peer carers/ buddies receive supervision?** |
| Yes ☐ No ☐ |
| **If yes, please describe (e.g. who provides the supervision? How often does it take place?)** |
| Comment |

| **How many prisoners in your establishment (i) received an assessment and (ii) were eligible for social care services between 1^st^ July 2022 and 30^th^ September 2022?** Please provide the exact numbers if known. If not, please provide working estimates followed by ‘Estimate’ or write ‘Not known’. | |
| --- | --- |
| Number of prisoner assessments | Number of eligible prisoners |

**SECTION 2: SOCIAL CARE OF WOMEN PRISONERS ON OR FOLLOWING RELEASE**

| **Please outline how your establishment is identifying prisoners with social care needs on or following release,** including (i) who undertakes this activity (e.g. social work staff; prison healthcare staff; prisoner orderlies) and (ii) how such prisoners are identified (e.g. via referral to intake/duty team on discharge). |
| --- |
| Comment |

| **In your opinion, how well are current measures to transfer prisoner assessments between authorities working?** |
| --- |
| Please Select |
| **Please describe any problems you are encountering with this process.** |
| Comment |

| **Please outline how your establishment works with the probation service to meet the social care needs of prisoners on release, including any partnership or joint commissioning arrangements you have.** |
| --- |
| Comment |

**SECTION 3: OVERVIEW**

| **Which of the following statements most closely describes the changes your establishment has made to meet its obligations to prisoners under the Care Act?** |
| --- |
| Please Select |

| **What are the main challenges your establishment is encountering in delivering the Care Act reforms for prisoners?** *Please tick three.* | | | | |
| --- | --- | --- | --- | --- |
| Greater than expected demand | ☐ | The additional costs to the local authority | | ☐ |
| Identifying eligible prisoners | ☐ | Coordinating care across agencies at ground level | | ☐ |
| Adapting community assessments for prisoners | ☐ | Coordinating care across agencies at a strategic level | | ☐ |
| Providing prisoner assessments | ☐ | Sharing information with other partners / agencies | | ☐ |
| Providing social care services in prisons | ☐ | Integrating prison and community-based services | | ☐ |
| Providing aids, equipment or adaptations in prisons | ☐ | Transferring information between local authorities | | ☐ |
| Reallocating or recruiting staff to meet new duties | ☐ | Training staff to deliver new duties | | ☐ |
| Providing advocacy services for prisoners | ☐ | Other | *Please specify* | ☐ |

| **Please use the following scale to indicate the extent to which you agree with the statements below:** *1=completely agree; 2=somewhat agree; 3=somewhat disagree; 4=completely disagree* | |
| --- | --- |
| The social care of prisoners in custody is good in this establishment | Number |
| The social care of prisoners on release is good in this establishment | Number |
| The implementation of the Care Act has improved the social care of prisoners in custody in this establishment | Number |
| The implementation of the Care Act has improved the social care of prisoners on release in this establishment | Number |

THANK YOU FOR COMPLETING THIS SURVEY
