# Supplementary material for: Women’s social care provision in prison has improved but challenges remain: findings from a national survey in England eight years after the 2014 Care Act
Source: BMC Public Health. 2026 May 1;26:1930. doi: 10.1186/s12889-026-27183-w (PMC13285503; doi:10.1186/s12889-026-27183-w)
Supplement: Supplementary file 4 — Supplementary Material 4. Topics covered in peer supporter training. [file 12889_2026_27183_MOESM4_ESM.docx]

**Supplementary Table S1. Potential topics covered in peer supporter training**

| **Survey** | **Topics covered** |
| --- | --- |
| LA #2 | Workbook 1: Understanding the role; duty of care; equality and diversity; working in a person-centred way; communication and advocacy; privacy and dignity  Workbook 2: Health and safety; handling information; cleaning and infection control.  Workbook 3: fluids and nutrition; safeguarding adults; assisting someone using a wheelchair; mental health; dementia and learning disabilities; health and healthy ageing. |
| LA #4 | Care Act basics  Referral pathways  Basic safeguarding  Boundaries  Roles and responsibilities  What to do if need to raise an issue |
| Governor #6 | Care Act training  Safeguarding |
| Governor #7 | Depends on what is required. E.g., if pushing a wheelchair, an awareness leaflet is issued. Helping to wash etc. - trained by healthcare |
